# A Research Agenda for Malaria Eradication: Monitoring, Evaluation, and Surveillance

**DOI:** 10.1371/journal.pmed.1000400

**Published:** 2011-01-25

**Authors:** The malERA Consultative Group on Monitoring, Evaluation, and Surveillance

## Abstract

Monitoring, evaluation, and surveillance measure how well public health programs operate over time and achieve their goals. As countries approach malaria elimination, these activities will need to shift from measuring reductions in morbidity and mortality, to detecting infections (with or without symptoms) and measuring transmission. Thus, the monitoring and evaluation and surveillance research and development agenda needs to develop the tools and strategies that will replace passive surveillance of morbidity with active and prompt detection of infection, including confirmation of interruption of transmission by detecting present and past infections, particularly in mobile populations. The capacity to assess trends and respond without delay will need to be developed, so that surveillance itself becomes an intervention. Research is also needed to develop sensitive field tests that can detect low levels of parasitaemia, together with strategies for their implementation. Other areas to explore include the rigorous evaluation of the utility of more detailed maps of disease and infection incidence and prevalence, the development of new maps to inform programmatic responses and the use of surveillance technologies based on cell phone or real-time internet Web-based reporting. Because any new strategies for monitoring and evaluation and surveillance for eradication have major implications for program implementation, research is also needed to test systems of delivery for acceptability, feasibility, efficiency, cost-effectiveness, and community engagement. Finally, there is a clear need to systematically review the information from past elimination efforts for malaria and other infectious diseases.

## Abstract 2

The Malaria Eradication Research Agenda (malERA) Consultative Group on Monitoring, Evaluation, and Surveillance present a research and development agenda for the tools required to monitor and evaluate progress toward malaria eradication.

**Summary Points**

- As countries approach malaria elimination, monitoring, evaluation, and surveillance activities will need to shift from measuring morbidity and mortality to detecting infections and measuring transmission
- Diagnostic tools (in particular, practical, field-ready tools for the detection of asymptomatic infection and DNA-based and serological biomarkers for malaria infection and transmission), and methods for tracking population movements will need to be developed and improved
- Development and use of better malaria distribution maps to guide elimination efforts requires more research
- Research is needed to assess and compare the performance of malaria transmission metrics at near zero transmission; new metrics will need to be developed for use in this setting
- Research should also be undertaken to test and improve the feasibility, efficiency, and cost-effectiveness of new information systems

## Introduction

Monitoring (the systematic tracking of program actions over time) and evaluation (the examination of progress and its determinants) activities measure how well public health programs operate over time and whether they are achieving their program milestones (markers of progress within and transition between phases) and ultimate goals. In the context of malaria program scale-up, monitoring and evaluation focuses on the evaluation of burden reduction, specifically morbidity and mortality [1]. However, as programs successfully reduce transmission to near-elimination levels, the measurement of malaria-associated morbidity and mortality burden becomes increasingly difficult and insensitive, particularly since a substantial proportion of infections will be asymptomatic in countries that experienced high infection rates in the recent past. Thus, burden measures that only detect clinical illness will not provide good estimates of ongoing transmission as countries approach elimination, and malaria program monitoring and evaluation and surveillance methods will need to focus on detecting infections (with or without symptoms) and measuring transmission dynamics as the primary indicators of interest.

The malERA Consultative Group on Monitoring, Evaluation, and Surveillance focused on defining the monitoring and evaluation and surveillance research and development needs as malaria elimination efforts unfold over the next 5–20 years. Information gaps and research needs were identified by the group by considering several broad thematic areas: lessons learned from countries that have recently achieved malaria elimination [2] or elimination of other diseases; the required evolution of the malaria monitoring and evaluation framework and indicators; surveillance as an intervention to reduce transmission; measurement of transmission interruption and maintenance of zero transmission; the tools (currently available and in the pipeline) needed, including diagnostics (screening, confirmation, and transmission measurement), mapping, and communication; and implementation issues. Information and research needs that were identified include: systematic reviews of existing information and experience, and assembly of that work into guidance; protocol or standards development for conduct of certain activities; and research and development activities to produce new information where guidance or experience does not exist, and new tools where these will enhance capabilities.

The World Health Organization (WHO) and the Roll Back Malaria (RBM) Global Malaria Action Plan (GMAP) characterize different “phases” of malaria control as programs progressively reduce transmission, though it is understood that these phases are part of a continuum rather than abrupt shifts [3],[4]. At high levels of transmission, initial efforts are focused on scaling up for impact (SUFI). Sustained control efforts subsequently lead to further transmission reduction. As very low levels of transmission are reached, programs move from a focus on control to a focus on pre-elimination and elimination, and finally prevention of reintroduction. Where appropriate, we shall indicate where proposed research and development activities would fit into this malaria elimination framework.

## Lessons Learned from Other Diseases or Current Malaria Elimination Programs

Several diseases other than malaria have been proposed for eradication or elimination. General lessons learned from these other disease elimination efforts have been summarized and underscore the critical role that monitoring and evaluation and surveillance play in these efforts [5]–[9]. The essential role of monitoring and evaluation and surveillance in informing elimination program efforts is particularly clear in past smallpox efforts and ongoing polio activities. Many countries have either eliminated or are in the process of pursuing malaria elimination. There is, therefore, a clear need to systematically review and summarize the monitoring and evaluation and surveillance lessons learned from both successful and unsuccessful disease elimination programs. In the context of malaria elimination, efforts are underway to summarize and disseminate recently accrued experience [2],[10]. This review work should be done even before the elimination phase.

General needs for monitoring and evaluation and surveillance that have already emerged from experience with elimination efforts for malaria and for other diseases include the need for: improved management of systems; improved identification of infected individuals; enhanced methods for engaging and developing community support; improved information sharing for advocacy (at the community level and involving high level leaders); and improved ways of conducting surveillance activities in the private sector. Past experience also indicates that current and future tools and strategies for monitoring and evaluation and surveillance will need to be tailored to the individual epidemiological, entomological, and socio-cultural situation.

## Monitoring and Evaluation Framework and Indicators

**Figure 1:**
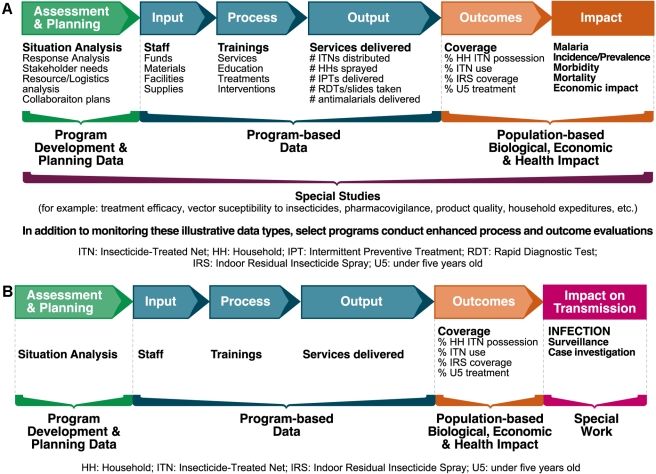
(A) Malaria monitoring and evaluation framework and illustrative data types. Source: adapted from [3]. (B) Evolving malaria monitoring and evaluation framework with emphasis on transmission. Image credit: Fusión Creativa.

The current Monitoring and Evaluation Framework for malaria comprises a series of activities, namely, Assessments and Planning, Inputs, Processes, Outputs, Outcomes (intermediate effects), and Impact (long-term effects; Figure 1A) [1]. Each part of this schema can be monitored with a specific set of indicators that tracks progress in program implementation. Historically, the malaria community has focused on illness and mortality reduction as indicators of impact, but will these and the other current indicators shown in Figure 1A serve us well for elimination efforts?

There is general consensus that these coverage indicators will continue to be useful because high intervention coverage will need to be maintained en route to elimination, especially in Africa where transmission is intense. However, as elimination is approached, other indicators will need to be adapted and new ones will need to be introduced. For example, indicators that track the proportion of cases with parasitological confirmation or that focus on coverage of individuals in specific geographic areas where foci of transmission are located will be needed. Similarly, if transmission blocking vaccines are deployed, coverage with the vaccine will need to be tracked. The utility of indicators and databases for parasite strain information that could differentiate indigenous from imported cases may need to be evaluated. In addition, methods and indicators for tracking population movements within and between countries and quantifying their contribution to the risk of malaria transmission may be useful. Furthermore, greatly reduced malaria morbidity and mortality levels (achieved through intervention scale-up and sustained control) will need to be monitored, although ultimately, as elimination approaches, the measure of impact will need to be infection and transmission (sometimes from introduced cases), and programs will need to include active case detection and case-based investigation and response within a revised Monitoring and Evaluation Framework (Figure 1B) (also see [11],[12]).

## Surveillance as an Intervention

As noted in the Introduction, monitoring and evaluation are critically required for measurement of malaria control program success. Over time, the term “surveillance” has become somewhat synonymous to some with monitoring and evaluation, but the WHO Global Malaria Eradication Program (GMEP), which lasted from 1955 to 1969, defined surveillance quite specifically as an integral action or intervention within that eradication program (Box 1) [13].

**Box 1. Definitions of Surveillance**

***Per conventional use:*** Surveillance is the ongoing, systematic collection, analysis, and interpretation of data, often incidence of cases of disease or infection. Surveillance data are used to plan, implement, and evaluate the progress in public health programs.

***Per the WHO Global Malaria Eradication Program:*** In malaria eradication terminology, surveillance was that part of the program aimed at the discovery, investigation, and elimination of continuing transmission, the prevention and cure of infections, and the final substantiation of claimed eradication. The individual functions of surveillance are case detection, parasitological examination, antimalarial drug treatment, epidemiological investigation, entomological investigation, elimination of foci by either residual spraying or mass drug administration, case follow-up, and community follow-up. In this definition, surveillance is seen as an intervention [16].

Malaria programs contemplating an elimination strategy must be prepared to change their strategies of monitoring and evaluation and surveillance as transmission is reduced [14],[15]. Thus, many countries begin scale-up of malaria control interventions with relatively high levels of malaria transmission and develop monitoring and evaluation programs that rely on the collection of routine information (often from health facilities and health management information systems) and on periodic population-based surveys. Together, these approaches collect information on intervention coverage and use as well as changes in malaria burden, but, as transmission intensity drops to near elimination levels, surveillance as defined by GMEP needs to increase (Table 1).

**Table 1:** Program activities and methods for transmission reduction in populations.

Potential Activity	Description and Purpose
Prevalence surveys	Usually population-based surveys to stratify risk, evaluate impact of interventions, and track progress towards elimination
Active case detection	Regular efforts to ascertain fever and infection in the community
Focused screening for infections (“active infection detection”)	Targeted search for main sources of rare cases (of *Pf*, *Pv*, drug-resistant *Pf*) and eliminating them
Case investigation	Detecting infections/cases around index cases for response
Mass screening and treatment	Screening large segments of the population to find and treat cases
Mass drug administration^a^	Administration of treatment to large segments of the populations regardless of infection status to reduce infections in a population with a relatively high infection rate
Surveillance for drug-resistant parasites	Enrollment of cases and follow-up of presence, density, or absence of parasites for in vivo resistance surveillance to assess treatment efficacy
Detection of gametocytaemia^b^	Find infections that contribute to ongoing transmission so that they can be treated to reduce transmission
Confirmation of elimination/detection of reintroduction^c^	Measurement of ongoing infection and transmission through sampling and use of biomarkers such as DNA or serology
Border screening/transit screening^d^	Rapid diagnostic testing of people crossing borders to allow immediate treatment of positives

a Note that mass drug administration is controversial for a variety of reasons but is presented here for completeness sake as it has been used to some benefit in the past (see also [25]).

b See also [11] and [25].

c See also [11] and [44].

d See also [12].

*Pf, P. falciparum*; *Pv, P. vivax.*

In the context of malaria elimination programs, the goal is to achieve complete reporting of each case of infection to health authorities, regardless of whether symptoms of fever or illness are present. Critically, malaria control programs usually identify individuals with fever/symptoms and laboratory-confirmed malaria parasite infection as “malaria cases,” but do not systematically assess the extent of asymptomatic malaria infection. As transmission decreases and individuals have less exposure to malaria, they lose acquired immunity and a higher proportion of infections present with symptoms. However, in populations in rapid transition from high exposure to low exposure, the proportion of persons with enough acquired immunity to harbour asymptomatic infections may remain substantial [16]. For example, in a low transmission setting in the western Pacific, >80% of infections identified in a recent cross-sectional population-based survey were afebrile [17]. Because asymptomatic infections are a reservoir of transmission to others, it is critical to seek all infections rather than just symptomatic cases as a method to reduce transmission.

For surveillance, standardized definitions for case/infection reporting are needed, along with a strong mandate for notification to health authorities of all malaria cases/infections in both public and private settings [18]. An important area for further research, therefore, is to investigate how tools such as legal requirements, financial inducements, and other novel approaches can be used to improve the coordination of detection and reporting of infections from the private sector to public health authorities. Importantly, all malaria cases/infections must be epidemiologically investigated, and linked to geographic and laboratory data (species and genotyping) so that the source and potential spread of infection can be quickly addressed.

Furthermore, reporting systems must be able to analyze reported data rapidly to assess trends over time and place, particularly as transmission drops and cases of infection become increasingly focal in distribution [19]. Although some control programs in endemic areas have malaria early warning systems, these systems need better performance characteristics (for example, better linkages with local information systems) before they can be truly useful in malaria elimination.

Assuming that effective infection detection and prompt and timely reporting exist, it is crucial that surveillance systems respond effectively to detected foci of infections and ultimately to individual infections in order to reduce transmission to a reproduction rate (*R*_0_) of <1. Although many programmatic responses to detected infections exist, there is neither a systematic description of such responses nor a well-defined evidence base to suggest the optimal strategic approach. For surveillance to be effective as an intervention, research on useful and efficient modes of both detection and response must be undertaken [20]. At the most basic level, it is currently unclear when programs transitioning to very low transmission conditions should add active case and infection detection to their response strategies, and whether additional vector control interventions are needed [21]. The evolution of these actions and the optimal sequence and mix needs further evaluation as is also discussed in the malERA paper on modeling [22].

Finally, countries embarking on malaria elimination must establish a system for continuous data validation to identify problems and to prepare for the process of certification of elimination [23],[24]. The concept of “good surveillance practices” should be implemented early to facilitate evaluation of the quality of the surveillance programs in the process of certification. Any system needs to be responsive and iterative to improve surveillance.

## Tools to Improve the Efficacy and Efficiency of Malaria Elimination

The overall strategic approach and mix of actions to address transmission is critical, but the identification and development of key tools and actions to optimize these strategic actions is equally important. Improved diagnostics for screening and surveillance, optimal use of drugs to reduce transmission [25], better mapping and use of mapping to track foci of infections, and improved communications for timely sharing of information and response are all important.

### Diagnostics

Tests that are sensitive enough to detect asymptomatic infections (as opposed to symptomatic infections or cases) are needed for elimination [26]. Ultimately, for simplicity and efficiency, it will be preferable to have the same test for both surveillance and case management. Elimination has already been achieved in some areas of low endemicity using currently available diagnostic tools (principally microscopy), but future efforts will include areas of previously high transmission that have achieved significant reductions through intervention scale-up. Existing diagnostic tools will need to be improved to achieve elimination in these more challenging transmission areas. Microscopy has some limitations in human resource capacity needs, sensitivity and ease of widespread use at the community level. Similarly, currently available rapid diagnostic tests (RDTs) have limited sensitivity compared with PCR, and need to be improved in terms of specificity, ease of use, cost, shelf stability under tropical conditions, *Plasmodium vivax* detection, ability to return to negative after treatment, and multispecies detection capacity where this is an issue [27]. As discussed in the malERA paper on Diagnoses and Diagnostics [11], rapid techniques not requiring blood sampling could provide major breakthroughs.

There is also a need to address issues around effective supervision and support. In particular, as transmission decreases, residual foci of infection may cluster in difficult-to-access populations that are underserved and less likely to access the health system. Strategies need to be developed and tested for improving access to and tracking of these populations for screening and surveillance of infection.

Finally, for eradication, diagnostic tools to measure transmission and its interruption will be critical. There is considerable interest in refining current serological tests (ELISA) to assist in the diagnosis of recent infection (incidence). Serology and other potential biomarkers are discussed in more detail below.

### Mapping and Stratification

Maps of the global distribution of *P. vivax* and *Plasmodium falciparum* that were generated by the Malaria Atlas Project have recently been published, but there is little research on how best to use these maps in the context of elimination [28],[29], and current mapping initiatives are limited by data availability, especially for scenarios that require high resolution. Maps can help define which low transmission areas are possible elimination targets, and can define the limits of adverse conditions for transmission, such as aridity and temperature. Maps can also help to determine where additional survey work is necessary for better spatial resolution of endemicity.

On a global scale, mapping malaria distribution will allow stratification to inform decision making and allow for interventions to be targeted or prioritized [29],[30]. When allied with modeling, such maps can indicate which combinations of interventions may be most appropriate and how much these will cost [22],[31]. However, for optimal utility, maps will need to be sensitive to different ecological scenarios and should provide enough detail of the principal factors governing transmission. From a technical point of view, more detailed maps are feasible, and linking mapping databases with other technologies such as Google will increase ease of access to mapping information.

For maps at regional or national levels, the spatial resolution of the information required is greater than that required for global scale risk mapping. Integration of mapping activities with the outputs of surveillance systems and other data sources (for example, intervention coverage and vector distribution) can provide the level of detail required to support effective elimination efforts. However, the incorporation of existing techniques for rapid mapping and the development of methods for optimal information dissemination to all levels of the malaria control programme remain major research challenges, as does the need to update protocols that do not currently incorporate our ability to image, map, and display information remotely, technologies that have been revolutionized since the Global Malaria Eradication Program.

As we progress closer to the goal of elimination, finer scale mapping will be required to identify residual foci [32]. Geographical reconnaissance remains part of control and elimination attempts in many countries and relies on local knowledge to make largely hand-drawn maps of potential foci and known vector breeding sites. This approach needs to be modernized to include a simple, user-friendly, and consistent methodology for micro-mapping. High resolution satellite imagery can detect households and water bodies at unprecedented spatial resolutions and thus replace some of the logistic burden in reconnaissance required to support elimination activities [33]. The use of maps to help find rare events such as individual cases of malaria is also a very poorly developed area that needs further research. Efficient signatures of transmission hotspots or disease foci (environmental, entomological, and human) are also not well known, so a final challenge will be to integrate novel monitoring and evaluation metrics with the existing mapping suite.

### Communication Technologies

Technological advances in communications and reporting systems (data collection, aggregation, and dissemination) offer potential improvements for surveillance in the context of elimination and eradication. Other prerequisites for good communication and reporting include basic health systems, and the capacity to analyze and use data to improve program performance. Most importantly, it is only the relevant and useful surveillance information that is required for prompt and timely communication.

Examples of potential enhancements to improve timely reporting include widespread implementation of cell phone technology [34], which has been used with considerable success in some areas such as Zanzibar and Madagascar to provide cluster detection and response [35]. Systems such as real-time internet Web-based reporting are also being explored. As noted above, the development of methods to integrate surveillance reporting technology with mapping tools is a priority. Critically, systems developed for collection, reporting, analysis, and dissemination of information must be structured so that they enhance decision-making and programmatic direction at the local (district) level. In addition, these systems must enhance the capacity of the program to provide useful and timely information to policy makers so that program status and progress towards elimination is clear and well explained [36].

### Resistance and High-Risk Populations

Tracking antimalarial drug resistance is an important activity in the context of malaria control, but it becomes less important in situations where there are relatively few cases who must all receive curative treatment. Thus, as elimination is approached, all outpatient therapy might be better administered as “directly observed therapy” as with tuberculosis. Because of inconsistent and inadequate access to health systems, difficult-to-access populations may be at increased risk of harbouring individuals with drug-resistant parasites. Strategies to improve access to these populations were discussed earlier (see also [25]).

As elimination is approached, declining transmission and thus fewer cases pose considerable challenges to monitoring for drug resistance because recruitment of sufficient numbers of patients is difficult and thus studies are prolonged and expensive. Simple drug efficacy protocols worked into routine surveillance activities at sentinel sites may be of some use; follow-up of all treated cases may be another approach to ensure that individuals have cleared parasites [37]. Molecular markers for resistance could be useful for population-level screening, although new assays relevant to current treatment drugs, particularly the artemisinins, need to be developed. Simple field PCR-based tools would be of use, both for resistance testing and to differentiate recrudescence from new infections [11].

Although no vaccine is currently available, it is likely that vaccines may be in use in the next decade. A challenge will be to monitor vaccines for efficacy against antigenically diverse parasites in the population, for their preventive effects against severe disease, and for their effects in settings with changing transmission, as well as for their effects on transmission itself (see also [38]). Newer molecular biology approaches may be useful in which human genes are used to predict immunological responses. Case control methodology can also be used to evaluate vaccine performance [39].

### Tools for Transmission Measurement: Metrics

Accurate measurement of malaria transmission is essential for monitoring and evaluation of malaria control programs that are approaching interruption of transmission and elimination. Past and present metrics for measuring malaria transmission in humans in endemic regions were recently systematically reviewed [14] and include: the proportion of individuals in a population with a palpable spleen (spleen rate); the proportion of individuals in a population with a laboratory-confirmed parasite infection per unit time (parasite rate [PR]); and the annual parasite incidence ([API], the product of the annual blood examination rate and slide positivity rate) [13],[14]. The entomological inoculation rate ([EIR], the number of infective bites per person per unit time) remains the gold standard measure of transmission.

A valid metric, or a combination of metrics, for measuring the interruption of transmission nationally or subnationally is critical as elimination is approached; but the existing metrics all have serious limitations when transmission is approaching zero, including the EIR, which is difficult, expensive, and virtually impossible to measure when there is very low transmission.

For example, API (or alternatively annual case incidence) is an important metric of transmission that can be obtained from routine surveillance reporting even when the PR falls below 5%. However, to ascertain API accurately, all cases in the population must be identified through comprehensive and complete surveillance of the target population, ideally using both passive and active detection. API ascertained through passive detection alone only records those symptomatic individuals who are captured through the routine surveillance system and would, therefore, provide a biased (too low) estimate of transmission for the entire target population. Additionally, its failure to detect individuals with asymptomatic infections in the population would critically hinder the clearance of parasites from human reservoirs when working towards elimination.

Similarly, to obtain an unbiased estimate of PR for a target population where the combination of passive and active detection is incomplete, probability sampling of the population is required (see next section also), but this is problematic when transmission is reduced to nonrandom residual foci of cases. Furthermore, using PR ascertained from probability biomarker surveys for validation of freedom from disease is challenging, with sample size and resultant uncertainty dependent on the probability of committing a type 1 and 2 error, the size of the population being sampled, and the sensitivity and specificity of the diagnostic test [40]. Thus, unless extremely large sample sizes are used, PR will provide imprecise measures at near zero transmission. Research is needed, therefore, to develop new metrics for transmission and to improve or modify data systems for these kinds of measurements.

### Tools for Transmission Measurement: Sampling and Surveys

To assess progress in intervention scale-up, nationally representative household surveys, such as the Malaria Indicator Survey (MIS), Demographic and Health Survey (DHS), and the UNICEF Multiple Indicator Cluster Survey (MICS), are recommended data collection instruments, Such surveys can provide population-based, relatively accurate, estimates of malaria intervention coverage, and parasite infection prevalence in the population, and should be useful in assessing sustained coverage of malaria interventions on a periodic basis, typically every 3–5 years.

However, once scale-up has been achieved and infection prevalence is approaching zero, or has been disrupted, such national surveys, with sample sizes typically of at least 2,000 households, would not be feasible for routine monitoring of low and/or focal malaria transmission. Alternative sampling methods for ascertaining population-based measures of malaria transmission are therefore needed. Ideally, such novel sampling strategies would approximate a “probability survey” (a survey having a known, nonzero probability of selection of all individuals for which it is desired to obtain estimates), while remaining logistically feasible to implement on a routine basis.

Once transmission has been interrupted, population-based collection of biological samples for detection of present infections, or serology for detection of past exposure and infection, could prove important for routine monitoring of populations, although improved assays will be required. Such approaches might include routine sampling of populations through antenatal clinics, immunization programs, and schools. Assessment of the validity of these new approaches for obtaining relatively unbiased population estimates will be needed.

To maintain interrupted transmission or elimination, malaria control programs need to be able to obtain representative and precise estimates of parasite exposure and present infections among mobile populations, especially those that frequently cross national borders. Although respondent-driven sampling (a sampling approach in which existing study subjects recruit future subjects from among their acquaintances) has been used for ascertaining point estimates among hidden populations, this approach would likely be inappropriate for monitoring malaria transmission among mobile populations. One approach that should be tested for routine monitoring of mobile populations is time location sampling (TLS), a variation of traditional two-stage cluster sampling in which the primary sampling units are time-location settings where mobile and/or hidden populations are known to congregate. Assessment of the accuracy of TLS estimates of parasite infection prevalence among mobile populations is needed, as well as cost-effectiveness in relation to other sampling methods.

### Biomarkers for Transmission Measurement

Serologic methods are currently an area of renewed interest as a potentially valuable tool for robust transmission measurement. Serology has been used to measure malaria exposure in humans for many years and was prominent in early elimination attempts [41],[42]. But, as these elimination attempts were scaled back, so was the use of serological characterization. With little use over several decades, these serologic assays lacked standardized, reproducible, and objective methods [43]. Recent technological improvements (for example, techniques that facilitate the production of antigens) mean that serology has now become a much more robust tool for transmission measurement [44]. However, there is a need to standardize protocols and antigens; currently there are many different methodologies with associated variation in results. Fundamental issues relating to the generation and maintenance of antibody responses in children and adults also need to be addressed.

Other research and development needs include the development of serological assays that are sensitive and specific for different *Plasmodium* species. Assays also need to be developed that show cumulative exposure to the parasite, as well as recent changes in transmission intensity by measuring both the prevalence and the magnitude of the antibody response. Serological methods might also be developed that distinguish between relapse and new infection with *P. vivax* by measuring exposure to mosquito saliva through the detection of antisaliva antibodies.

PCR or similar molecular amplification–based methods may also prove useful for the measurement of transmission reduction/interruption, especially if pooled sampling and high-throughput automated techniques are used to handle large numbers of samples [45]. There is limited experience to date with these methods as tools to measure transmission; further research may help to elucidate their potential.

For all biomarkers, the most desirable assays would not require blood sampling so research into biomarkers in saliva or other bodily fluids is needed. Finally, for all biomarkers, there is a need to develop criteria that define an area as “malaria free.”

## Concluding Remarks

The new strategies proposed in this paper by the malERA Consultative Group on Monitoring, Evaluation, and Surveillance for eradication have major implications for implementation, and research is needed to test best systems of delivery for acceptability, feasibility, efficiency, and cost-effectiveness. Box 2 draws our discussions together in the form of a research and development agenda for monitoring, evaluation, and surveillance.

**Box 2. Summary of the Research and Development Agenda for Monitoring, Evaluation, and Surveillance**

- Update the malaria monitoring and evaluation Framework to include transmission reduction, and develop key data elements for a surveillance system from a systematic review of previous elimination attempts
- Systematically review lessons learned from experiences with surveillance as an intervention to determine how it can be tailored to various programmatic settings
- Identify appropriate program time points for introduction of malaria infection detection in active or passive modes
- Develop improved diagnostic tools for use in monitoring and evaluation and surveillance, focusing on practical field-ready tools for detection of asymptomatic infection
- Develop information systems to monitor malaria infections, facilitate timely local program decisions and responses to reduce transmission
- Develop methods, indicators, and shareable databases for parasite strain information to better track transmission
- Develop methods for accessing and tracking population movements and quantifying their contribution and risk of malaria transmission
- Explore how maps can be constructed to: show the probability of a threshold of transmission being exceeded; incorporate a wider range of metrics such as serological and entomological data; assess cost-effectiveness of national stratification initiatives based on remotely sensed satellite data
- Perform a systematic review to assess and compare metrics of malaria transmission at near zero transmission levels; research the validity of novel metrics to measure transmission at near zero levels, and to measure transmission potential within areas where transmission has been eliminated
- Assess the precision, bias, feasibility, and cost-effectiveness of novel sampling methods for routine monitoring of present and past infections in target populations, including mobile populations
- Conduct research to develop biomarkers such as DNA-based methods or serology as monitoring and evaluation and surveillance tools

## Abbreviations

API: annual parasite incidence
PR: parasite rate
